# SLC38A9: A lysosomal amino acid transporter at the core of the amino acid-sensing machinery that controls MTORC1

**DOI:** 10.1080/15548627.2015.1091143

**Published:** 2015-10-02

**Authors:** Manuele Rebsamen, Giulio Superti-Furga

**Affiliations:** a CeMM Research Center for Molecular Medicine of the Austrian Academy of Sciences, Vienna, Austria; b Center for Physiology and Pharmacology, Medical University of Vienna, Vienna, Austria

**Keywords:** amino acid transport, cancer, metabolism, MTOR, nutrient sensing, solute carrier proteins

## Abstract

The mechanistic target of rapamycin (serine/threonine kinase) complex 1 (MTORC1) acts as a crucial regulator of cellular metabolism by integrating growth factor presence, energy and nutrient availability to coordinate anabolic and catabolic processes, and controls cell growth and proliferation. Amino acids are critical for MTORC1 activation, but the molecular mechanisms involved in sensing their presence are just beginning to be understood. We recently reported that the previously uncharacterized amino acid transporter SLC38A9 is a member of the lysosomal sensing machinery that signals amino acid availability to MTORC1. SLC38A9 is the first component of this complex shown to physically engage amino acids, suggesting a role at the core of the amino acid-sensing mechanism.

MTORC1 activation at the surface of lysosomes depends on the integration of multiple inputs that signal through 2 parallel pathways. Growth factors and energy levels affect MTORC1 by regulating the tuberous sclerosis complex (TSC) and RHEB activity, whereas amino acid-dependent control of MTORC1 relies on a lysosomal multicomplex machinery comprising the v-ATPase, the Ragulator complex and RRAG GTPases. The Ragulator complex tethers to the lysosome the RRAG GTPase heterodimers, which, in the presence of amino acids and in their active nucleotide loaded status, recruit MTORC1 to the lysosomal surface where it is activated by RHEB. Amino acid signaling through the Ragulator and the RRAG GTPases requires the v-ATPase, but the mechanism(s) of the sensing has been unclear.

We discovered that the lysosomal amino acid transporter SLC38A9 is a physical and functional component of this sensing complex. Several plasma membrane amino acid transporters influence MTORC1 activity by modulating intracellular amino acid levels, so we hypothesized that an amino acid transporter might also be involved at the lysosomal level. Based on expression and localization data, we focused our attention on SLC38A9, an uncharacterized member of the amino acid transporter solute carrier family 38. Tandem affinity purification coupled to gel-free liquid chromatography tandem mass spectrometry (LC-MS/MS) using SLC38A9 as bait identified as specific interactors the 5 LAMTOR proteins that form the Ragulator complex as well as the 4 RAG GTPases (RRAGA, RRAGB, RRAGC, and RRAGD). Binding of endogenous SLC38A9 to the Ragulator-RRAG GTPase complex was confirmed in multiple cell types, whereas none of the other related amino acid transporters tested, SLC38A1, SLC38A2, SLC38A7, SLC36A1, and SLC36A4, showed any affinity to this complex. Supporting the notion that SLC38A9 is an integral and probably stoichiometric part the complex, proteomic analysis using 4 LAMTORs, RRAGA and RRAGC as baits revealed that SLC38A9, together with RPTOR, was the only interactor identified in all these purifications. The N-terminal cytoplasmic region of SLC38A9 is sufficient and required to mediate interaction. In contrast to the transmembrane transporter region that shares sequence similarity with the other members of the SLC38A family, the N-terminal portion is characteristic of SLC38A9, and mutation of evolutionarily conserved motifs in this region disrupts binding capacity toward the Ragulator-RRAG GTPase complex.

Given that SLC38A9 belongs to an amino acid transporter family, but was still completely uncharacterized, we investigated its amino acid transport competence in vitro by reconstituting liposomes with recombinant SLC38A9. Transport assays in proteoliposomes showed transport activity for glutamine and arginine that, together with leucine, are the main amino acids that are important for MTORC1 activity. Consistent with its role as a lysosomal transporter, SLC38A9 shows higher Gln transport activity at acidic pH and is more efficient in promoting efflux from a reconstituted proteoliposome than influx. These results indicate that SLC38A9 is a low-capacity amino acid transporter, and, importantly, the first member of the lysosomal amino acid-sensing complex shown to physically engage amino acids.

Taking advantage of RRAG GTPase nucleotide binding mutants mimicking either the GTP or GDP loaded status, we observed that binding of SLC38A9 is tighter to RRAG GTPase mutants mirroring amino acid starvation conditions. Accordingly, amino acid starvation results in increased RRAGC binding to SLC38A9 that is reverted by amino acid replenishment. This is not observed when the N-terminal cytoplasmic tail of SLC38A9 is used, indicating that it is the transmembrane amino acid-binding portion that confers amino acid sensitivity.

Stable overexpression of SLC38A9 results in sustained MTORC1 activation upon amino acid starvation. This is reflected in prolonged phosphorylation of its substrate ULK1 and delayed induction of autophagy as well as TFEB nuclear translocation. Strikingly, expression of the N-terminal cytoplasmic region is sufficient to support MTORC1 activity; this demonstrates that the transmembrane transporter portion is dispensable in this context to confer the gain-of-function phenotype. Conversely, SLC38A9 silencing results in an impairment of amino acid-induced MTORC1 activation, further supporting its role as positive regulator of MTORC1. v-ATPase inhibition by concanamycin A blocks amino acid-induced MTORC1 activation, but does not affect the sustained signaling induced by SLC38A9 overexpression, suggesting that these components act in concert to signal amino acid sufficiency to MTORC1.

Together, our results and those from a concomitant study by David Sabatini and colleagues, and further confirmed recently by work from Christian Behrends and colleagues, highlight SLC38A9 as a component of the lysosomal amino acid sensing machinery that controls MTORC1 activation (Fig. 1). Considering the low-capacity transport activity and the observation that the N-terminal cytoplasmic domain is sufficient for binding and gain of function, we propose a model in which SLC38A9 acts as a transceptor (transporter-receptor) whose function is not exclusively mediated by the bulk transport of amino acids, but in which amino acid binding/transport results in allosteric signal transduction by affecting SLC38A9 interaction with other members of the complex and in particular the RRAG GTPases. A similar transceptor mode-of-action has been described for several nutrient sensors, such as the yeast amino acid sensor Ssy1.

**Figure 1.**
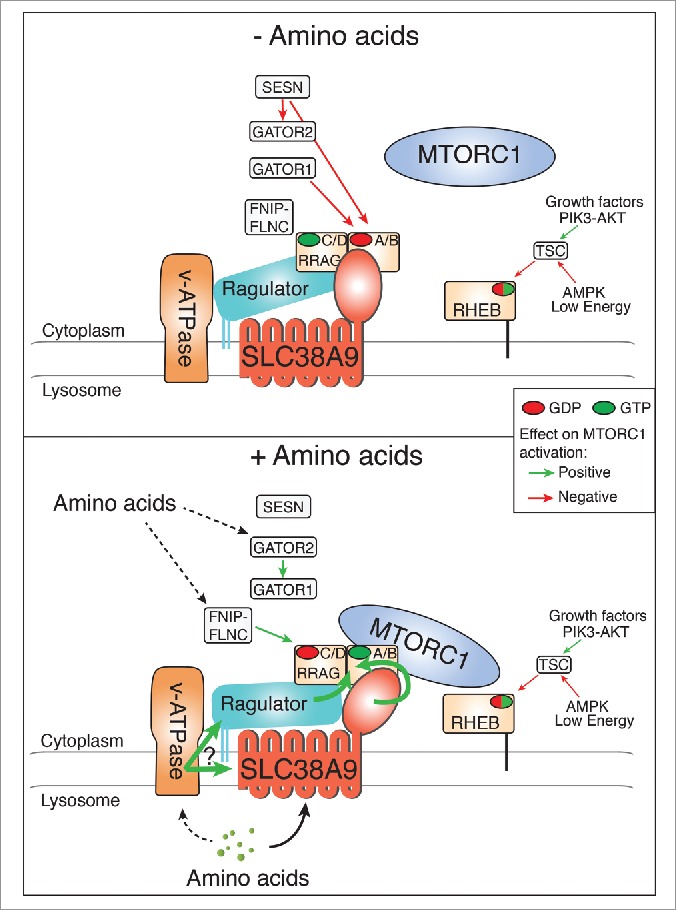
SLC38A9 is an integral component of the lysosomal amino acid-sensing machinery required for MTORC1 activation. Model of SLC38A9 function: direct amino acid binding/transport results in allosteric regulation of SLC38A9, affecting its interaction with the RRAG GTPases and contributing to MTORC1 activation. Other key regulators of MTORC1 are also shown. Arrows indicate regulation and colors the overall positive (green) or negative (red) effect on MTORC1 activity.

Our results, together with work from others identifying novel regulators of MTORC1 activation in dependency of amino acid availability, start to unravel the complexity of this signaling process and make clear that multiple sensing mechanisms are likely to exist, probably displaying different specificities in terms of amino acid detection and localization. Thus, SLC38A9 may be the first of a series of components remaining to be identified. Many intriguing questions remain, including the mechanism by which the active RRAG GTPase heterodimer recruits the cytoplasmic MTORC1 to the lysosomes and which additional regulatory steps may be required to mediate full activation of MTORC1 once at the lysosomes. Future studies should also clarify how these regulatory inputs are integrated and reveal their specificity and redundancy, in particular in physiological situations where amino acid availability could be limiting.

